# Engineering Mitochondriotropic Carbon Dots for Targeting Cancer Cells

**DOI:** 10.3390/ph14090932

**Published:** 2021-09-16

**Authors:** Archontia Kaminari, Eleni Nikoli, Alexandros Athanasopoulos, Elias Sakellis, Zili Sideratou, Dimitris Tsiourvas

**Affiliations:** 1 National Centre for Scientific Research “Demokritos”, Institute of Nanoscience and Nanotechnology, 15310 Aghia Paraskevi, Greece; a.kaminari@inn.demokritos.gr (A.K.); h.nikoli@inn.demokritos.gr (E.N.); e.sakellis@inn.demokritos.gr (E.S.); z.sideratou@inn.demokritos.gr (Z.S.); 2 National Centre for Scientific Research “Demokritos”, Institute of Biosciences and Applications, 15310 Aghia Paraskevi, Greece; alexandr@bio.demokritos.gr

**Keywords:** carbon dots, mitochondrial targeting, bioimaging, cancer cells, membrane potential

## Abstract

Aiming to understand and enhance the capacity of carbon dots (CDs) to transport through cell membranes and target subcellular organelles—in particular, mitochondria—a series of nitrogen-doped CDs were prepared by the one-step microwave-assisted pyrolysis of citric acid and ethylenediamine. Following optimization of the reaction conditions for maximum fluorescence, functionalization at various degrees with alkylated triphenylphosphonium functional groups of two different alkyl chain lengths afforded a series of functionalized CDs that exhibited either lysosome or mitochondria subcellular localization. Further functionalization with rhodamine B enabled enhanced fluorescence imaging capabilities in the visible spectrum and allowed the use of low quantities of CDs in relevant experiments. It was thus possible, by the appropriate selection of the alkyl chain length and degree of functionalization, to attain successful mitochondrial targeting, while preserving non-toxicity and biocompatibility. In vitro cell experiments performed on normal as well as cancer cell lines proved their non-cytotoxic character and imaging potential, even at very low concentrations, by fluorescence microscopy. Precise targeting of mitochondria is feasible with carefully designed CDs that, furthermore, are specifically internalized in cells and cell mitochondria of high transmembrane potential and thus exhibit selective uptake in malignant cells compared to normal cells.

## 1. Introduction

Carbon dots (CDs), an emerging group of fluorescent nanomaterials with typical sizes of less than 10 nm, are similar to semiconductor quantum dots with respect to their unique optical and electrochemical properties while, more importantly, their preparation is environmentally friendly and they exhibit water solubility, chemical inertness, low to minimal toxicity and biocompatibility [1]. Due to their intrinsic fluorescence properties and their ability to encompass functional groups on their surfaces, a variety of applications ranging from sensing and catalysis [2,3,4] to drug and gene vectors [5,6] or for biological imaging [2,7,8,9] are being intensively studied. Specifically, the presence of reactive groups on their surfaces enables their functionalization with biologically active compounds and/or targeting groups, while, on the other hand, their intrinsic fluorescence properties coupled with their minimal cellular toxicity and targeting ability make them suitable as diagnostic agents. Thus, these nanoparticles are considered attractive “theranostic” agents [10], i.e., agents that can be used for the simultaneous targeted therapy and diagnosis of diseases, with a special emphasis on cancer [5], and are in an advantageous position compared to other promising, but less safe, carbon-based nanovectors, such as carbon nanotubes or graphene/graphene oxides [11,12].

In addressing the fight against different forms of cancer, research has recently focused, among other aspects, on the mitochondrial function of malignant cells. This is due to the continuous recognition of mitochondrial dysfunctions as the underlying causes of a number of pathologies, including cancer [13,14], which has resulted in the rise and growth of the so-called mitochondrial medicine [15,16]. Drug delivery to mitochondria requires the development of mitochondriotropic drug delivery systems, which has been pursued by attaching mitochondriotropic ligands in various drug nanocarriers, such as liposomes, polymers, dendrimers or carbon dots [17,18,19,20,21,22,23].

The vast majority of mitochondriotropic ligands belong to the family of delocalized lipophilic cations (DLCs), the most prominent of them being the triphenylphosphonium group (TPP), a non-toxic, positively charged chemical moiety, attached in most cases at the end of an alkyl chain (alkyl-TPP) [21,24,25,26,27]. Although charged species cannot passively cross biological membranes, the lipophilic character of alkylated TPP and the delocalization of its positive charge lead to the ability to cross both the cell membrane as well as the mitochondrial membrane. The cationic charge of TPP-decorated nanoparticles on one hand and the large negative mitochondrial membrane potential (Δψ_m_ = 150−180 mV) of the mitochondrial membranes on the other can result in up to several hundred fold mitochondrial uptake [24,27]. However, there are certain limitations as a number of factors are also at play [27,28], including the actual membrane potential, the presence of pH gradients, the pk_a_ of the vector and the presence of other subcellular organelles, especially lysosomes. The latter, due to their acidic interior (pH~5), are prone to accumulate cationic species within, a phenomenon referred to as “acid trapping” or lysosomotropism [29], which apparently competes with mitochondriotropism.

The advantages of carbon dots as promising theranostic agents, and the increasing awareness of the significance of mitochondria in several diseases, led to the development of TPP-functionalized carbon dots. Such nanoparticles could be employed either as novel mitotrackers, given their extremely high stability against photobleaching, or/and as drug carriers after further conjugation with anticancer agents. Several reports were based on the functionalization primarily of nitrogen-doped (N-doped) carbon dots, which are among the most widely studied CDs [30], because they utilize low-cost starting materials, such as organic acids and amines, are easily produced, have high quantum yields and, due to the presence of NH_2_ groups on their surface, can be easily conjugated to alkylated TPP groups [31,32,33]. However, mitochondrial localization is not always complete, as the CDs can also be located in lysosomes or in the cytosol, while the rather low fluorescence signal in confocal laser scanning microscopy experiments dictates the use of concentrations much higher than those typically used for already existing fluorescent mitochondriotropic agents. The latter can, in part, be responsible for their localization outside the mitochondria since there is obviously a certain upper limit in the capacity of mitochondria to host the mitochondrial vectors and, thus, the exclusivity of mitochondria accumulation is unlikely. The low fluorescent signal of this type of N-doped carbon dots, observed when they are inside a live cell, can be ascribed to the quenching of their fluorescence by various entities present in the cell’s interior, including metal ions (such as Fe(II), Cd(III), etc.) [1,2,7,34] and free radicals [22], or to the aggregation-induced quenching of the CDs upon interacting electrostatically with oppositely charged biopolymers [35]. Further to the above limitations, these CDs are characterized by negligible absorption in the visible and near-infrared region of the electromagnetic spectrum, which is of particular interest for, among others, live cell, ex vivo and in vivo imaging applications. To overcome this disadvantage, special, more complicated, synthetic approaches were employed for the preparation of CDs that both target mitochondria and absorb in the low to middle visible wavelength region [36,37,38].

Our group had previously validated the capacity of a variety of nanodrug delivery systems based on dendritic polymers to target mitochondria by the introduction of alkyl-TPP groups at their surface and was thus able to show the efficient delivery of drugs into the mitochondria of cultured cells, achieving cytotoxic efficacy at very low drug concentrations [39,40], or selectivity for cancer stem-like cells cultured in 3D cell cultures [41].

**Scheme 1:**
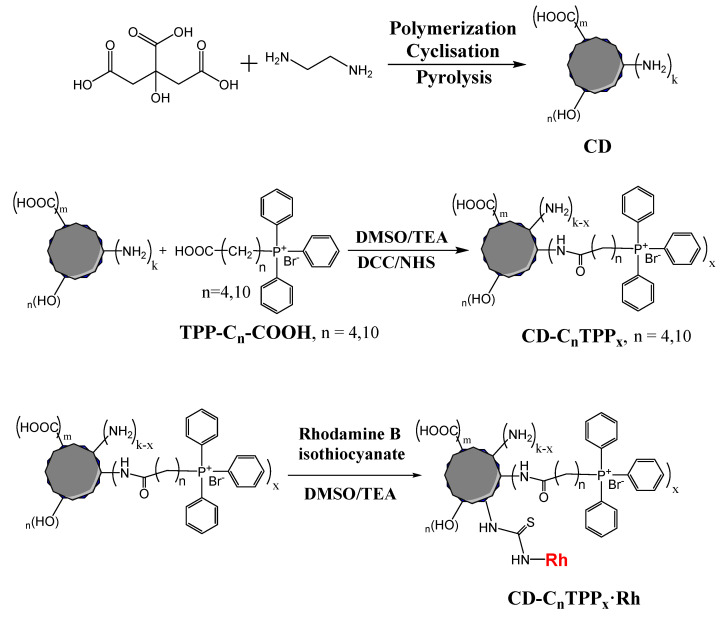
Microwave synthesis of N-doped carbon dots and their subsequent functionalization with alkyl-TPP groups and rhodamine B isothiocyanate.

The novelty of the present work refers to the engineering of biocompatible carbon dot–TPP fluorescence systems to target cell mitochondria for therapeutic and/or bioimaging purposes for diseases, such as cancer. To this end, N-doped carbon dots were initially synthesized by the microwave irradiation of citric acid and ethylenediamine, which is a fast and low-cost process for carbon nanoparticle synthesis [34,42,43,44,45,46]. After optimization of the synthetic process, the resulting CDs exhibit fluorescence with a very good quantum yield, non-toxicity and biocompatibility. Subsequently, the surface of the CDs was functionalized with different degrees of alkylated triphenylphosphonium functional groups (alkyl-TPP) of two different alkyl chain lengths (Scheme 1). Further functionalization with rhodamine B isothiocyanate was deemed necessary to improve the live cell imaging in the visible spectrum, significantly reduce the required amount of CDs employed in in vitro cell studies and clarify the effect of alkyl chains and degree of alkyl-TPP functionalization in achieving mitochondrial, instead of lysosomal, targeting. Thus, a highly efficient red-emitting CD derivative for optical imaging with almost excellent mitotropism was obtained, enabling its use as a mitotracker and as a potential apoptotic marker. Moreover, its cell internalization is dependent on cell membrane potential, rendering it suitable for discerning cancerous from normal cells.

## 2. Results

### 2.1. Synthesis of Nitrogen-Doped CDs and of Their Alkyl-TPP Derivatives

Nitrogen-doped CDs were produced by microwave-assisted polymerization, cyclization and pyrolysis of citric acid (CA) and ethylenediamine (EDA), according to already published protocols [47]. Under the experimental set-up employed, optimization of the reaction conditions with respect to the molar ratio between the two reactants and the reaction time was first performed to obtain the highest possible quantum yield (QY). An increase in the reaction time above a certain optimum value (in our case, 2 min) has a detrimental effect on the QY (Table S1, Supplementary Material), while the CA:EDA molar ratio also bears an effect on the QY (Table S2, Supplementary Material), with an optimum value of CA:EDA of 0.90. The CDs prepared under these optimum conditions had a QY of 48.5%. The primary amino groups of CDs were quantified by employing ninhydrin and found to be 1.18 ± 0.02 mmol ΝH_2_/g (Table S3, Supplementary Material).

The chemical structure of the nitrogen-doped CDs was initially established by ^1^H and ^13^C NMR spectroscopy. Specifically, in the ^1^H NMR spectrum (Figure S1A, Supplementary Material), the peaks in the range between 4 and 2 ppm were attributed to the protons connected to the saturated carbon atoms that are relative to the amino or amide groups or to oxygen-containing groups. The multiple peak at 6.0–5.5 ppm was assigned to the protons connected to the unsaturated carbons of the CD moiety, revealing that the reactions, which occurred during the microwave irradiation, were both polymerization reactions between the citric acid and ethylenediamine as well as cyclization reactions of the intermediates formed during the polymerization process [42]. Further evidence regarding the reactions that took place was obtained from the ^13^C NMR spectrum of nitrogen-doped CDs (Figure S1B, Supplementary Material). Specifically, the peaks in the region between 45 and 35 ppm were ascribed to aliphatic (sp^3^) carbons, the peaks at 74 and 72.5 ppm were assigned to saturated carbons relative to epoxy and hydroxyl groups, respectively, while the signals in the range of 170–150 ppm were attributed to double bond and aromatic carbon atoms. Finally, the peaks at 173–171 ppm and those in the region of 181–176 ppm corresponded to amide (NHCO) and carbonyl (C=O) carbons, respectively [34].

Alkyl-TPP functionalization was realized by the interaction of the primary amino groups of CDs with the carboxylic groups of (4-carboxybutyl)triphenylphosphonium bromide (C_4_TPP) or (10-carboxydecyl)triphenylphosphonium bromide (C_10_TPP). The various alkyl-TPP-functionalized carbon dots synthesized by employing carbon dots (100 mg) with various moles of C_4_TPP or C_10_TPP (10–25 mmol) are shown in Table S3, Supplementary Material. By determining the number of primary amino groups of each sample and taking into consideration the number of primary amino groups of the starting CDs, it was possible to gain a reasonable approximation of the degree of functionalization for each sample, which was later confirmed by NMR spectroscopy. Thus, we were able to obtain 30–80% degrees of functionalization (Table S3, Supplementary Material). It was, however, found that high degrees of functionalization, especially for the C_10_TPP-functionalized CDs, resulted in reduced water solubility and a small but significant increase in cytotoxicity (see below Section 2.3). Therefore, only the derivatives CD-C_4_TPP and CD-C_10_TPP with ca. 50% degree of functionalization were further studied in detail and subsequently used in in vitro cell studies.

The successful introduction of alkyl-TPP groups to CDs’ surfaces was confirmed by ^1^H NMR spectroscopy (Figure S2, Supplementary Material). Comparing the spectra of the alkyl-TPP-functionalized CDs to those of CDs, apart from the characteristic peaks of CDs, signals originating from the alkyl-TPP groups and the newly formed amide groups were observed. Specifically, in the ^1^H NMR spectra of both alkyl-TPP derivatives, the characteristic peaks in the region of 8.0–7.6 ppm, attributed to the aromatic protons of TPP groups, were observed. Furthermore, the presence of C_4_TPP on CDs was also confirmed by the peaks at 2.2, 1.7 and 1.6 ppm, assigned to α-, β- and γ-CH_2_ relative to newly formed amide groups. In the case of CD-C_10_TPP, the peaks centered (i) at 2.2 ppm were attributed to the protons of α-CH_2_ relative to the newly formed amide group, (ii) at 1.6 and 1.4 ppm assigned to β- and γ-CH_2_ relative to TPP groups, respectively, and (iii) at 1.1 ppm, ascribed to methylene protons of the aliphatic chain, revealed the successful attachment of C_10_TPP on the CD surface [40]. To determine the alkyl-TPP content in CD-C_4_TPP and CD-C_10_TPP employing qualitative NMR (qNMR), ^1^H NMR spectra were also acquired using maleic acid as an internal standard. Thus, from their ^1^H NMR spectra, by comparing the integrals of the peaks at (i) 6.35 ppm attributed to the protons of methine groups of maleic acid and (ii) 8.0–7.6 ppm assigned to the aromatic protons of TPP groups, one can calculate that, on average, 0.51 mmol (0.21 g) C_4_TPP or 0.57 mmol (0.29 g) C_10_TPP groups were conjugated to 1 g of CD-C_4_TPP and CD-C_10_TPP, respectively. Taking into consideration the number of primary amino groups per g of N-doped CDs (1.18 mmol ΝH_2_/g), the corresponding degrees of functionalization were 54% and 49%, respectively, in very good agreement with the results obtained by the quantification of the amino groups of the same derivatives employing the ninhydrin method (Table S3, Supplementary Material). Finally, these two derivatives were further tagged with rhodamine B, affording the corresponding CD-C_4_TPP•Rh and CD-C_10_TPP•Rh derivatives. The rhodamine degree of functionalization, as determined with UV–Vis spectrophotometry, was kept low (0.4% *w/w*) in order to not significantly affect the cell internalization properties of the CDs.

### 2.2. Characterization of Nitrogen-Doped CDs and of Their Alkyl-TPP Derivatives

FTIR spectroscopy was also used to elucidate, in conjunction with NMR, the chemical structure of CDs and to follow the gradual increase in the CDs’ degree of functionalization with the alkyl-TPP groups. The FTIR spectra of the N-doped CDs (Figure S3, Supplementary Material) were characterized by a broad peak centered at 3300 cm^−1^, attributed to the stretching vibrations of O-H and N-H groups, and of a peak at 3080 cm^−1^, the overtone of the amide II band of the secondary amide groups formed during the initial polymerization step. The peak at 2950 cm^−1^ was due to the stretching band of CH_2_ groups, while the peak at 1705 cm^−1^ was attributed to the C=O vibration of the carboxylic groups. The amide I, II and III bands were present at 1650, 1545 and 1230 cm^−1^ respectively, while the CH_2_ bending vibrations appeared at 1435 cm^−1^. The stretching vibrations of the C=C groups or of the C=N groups were both overlaid under the 1650 cm^−1^ band. The band at 1400 cm^−1^ was attributed to the C-O-H in plane bending, and the band at 1075 cm^−1^ was attributed to both the stretching C-O band of hydroxyl groups or oxidized carbons, as well as to the C-N stretching of primary amines. In the infrared spectra of functionalized CDs, the bands of the alkyl-TPP moieties were also present (Figure S3, Supplementary Material). The CH_2_ stretching vibration bands of the alkyl chains were registered at 2935 and 2857 cm^−1^, and their bending and rocking vibrations at 1435 and 724 cm^−1^, respectively. Additionally, the aromatic rings of the TPP group were evident by the bands at 1435 (ν CC), 752 (γ CH) and 692 (φ CC) cm^−1^, while the bands at 1115, 511 and 499 cm^−1^ were assigned to P-C stretching vibrations (X-sensitive modes) [40,48]. The ζ-potential of the N-doped CDs at a pH of 7.4 (phosphate buffer) was found to be 2.5 ± 1.2 mV, suggesting that, at this pH level, the nanoparticles, due to the presence of both carboxylic and amine groups (cf. Scheme 1), were close to charge neutrality. The addition of C*_n_*TPP moieties increased the charge, as expected, leading to ζ-potential values of 6.2 ± 1.8 mV for CD-C_4_TPP•Rh and 5.8 ± 1.6 mV for CD-C_10_TPP•Rh. The observed increases were not prominent, given that the introduction of a positively charged TPP moiety resulted in the sequestration of an amino group that also had a positive charge contribution.

**Figure 1:**
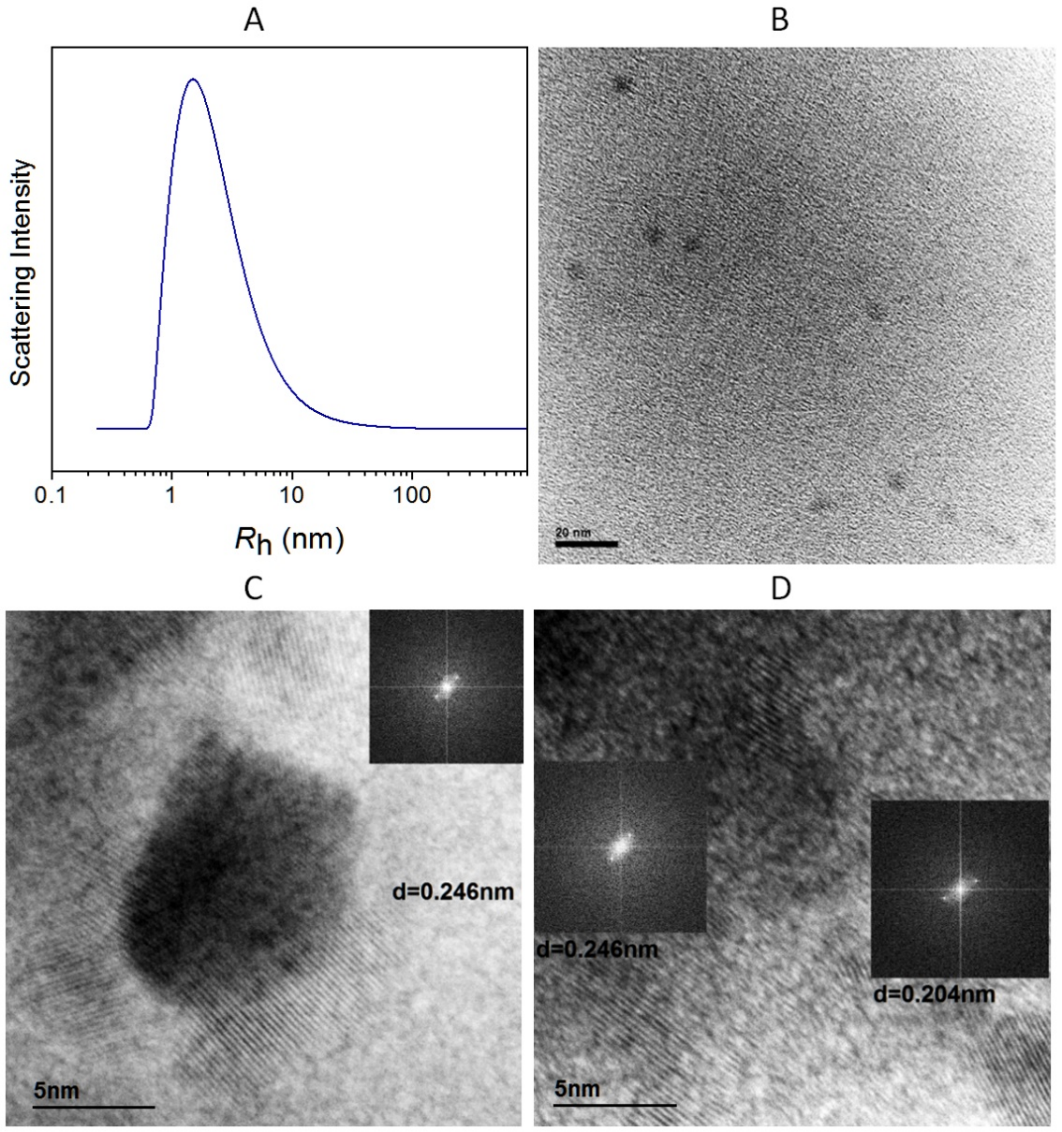
(**A**) Hydrodynamic size distribution of N-doped CDs. (**B**) TEM bright-field images of N-doped CDs; scale bar: 20 nm. (**C**,**D**) High-resolution TEM (HRTEM) images of N-doped CDs. The insets are the corresponding fast Fourier transform (FFT) patterns.

The hydrodynamic radii of CDs in water were determined by employing dynamic light scattering (DLS) to be centered at around 2 nm, with a narrow size distribution (Figure 1A). The DLS results were confirmed by TEM bright-field images (Figure 1B, and Figure S4, Supplementary Material) revealing the presence of mono-dispersed, quasi-spherical, well-separated nanoparticles, with diameters ranging between 3 and 6 nm. High-resolution transmission electron microcopy (HRTEM) was further used for a detailed characterization of the morphology and structure of CDs. As shown in Figure 1C,D and in Figure S4, Supplementary Material, the images reveal the presence of crystalline layers, as well as amorphous regions. The crystalline areas displayed lattice fringes with spacings of 0.246 nm, 0.220 nm and 0.204 nm that were consistent with the lattice constant α (2.462 Å), the d_100_ spacing (2.132 Å) and the d_101_ spacing (2.032 Å) of graphite, respectively [49,50,51]. The observed lattice spacing of 0.317 nm was slightly smaller than expected (d_002_ = 3.355 Å). Such smaller than expected values in carbon dots have also been reported previously [6,52,53]. In analogy with the strong 3.1 Å diffraction line that was observed in certain carbon allotropes, this can be tentatively attributed to the presence of a compressed (strained) graphitic structure [54]. In line with the above, their X-ray diffractogram (Figure S5, Supplementary Material) consisted of very broad peaks originating from both the amorphous and nanocrystalline regions. The peaks centered at around 27° (d≈3.3 Å) and 42° (d≈2.15 Å) corresponded, respectively, to the (002) and (100) lattice spacings of the graphitic structure [30,49]. Overall, these findings are in line with similar studies on heteroatom-doped CDs that also show weak and broad peaks due to the introduction of such heteroatoms in their structure [30].

**Figure 2:**
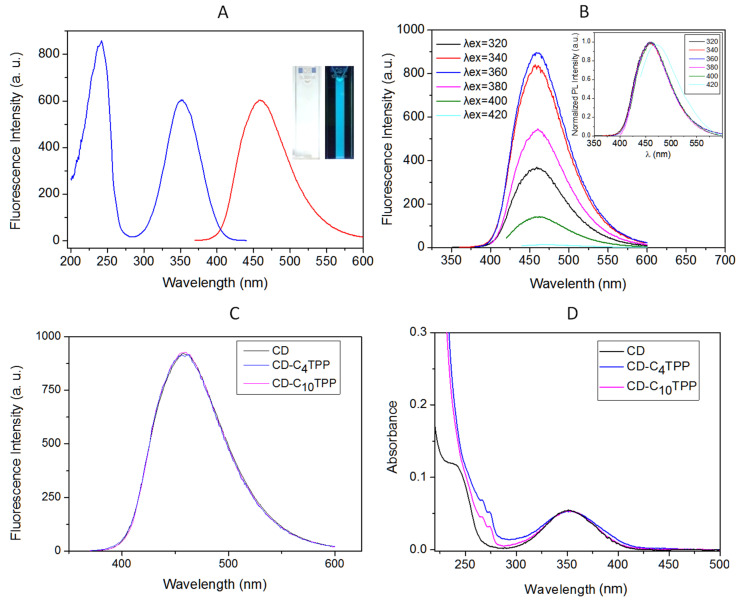
(**A**) Excitation (blue line) and emission spectra (red line, λ*_ex_* = 352 nm) of N-doped CDs in water. Inset: photographs of their solutions under ambient light (left) and under a 365 nm UV lamp (right). (**B**) Emission spectra at progressively increasing excitation wavelengths (320 nm–420 nm). Inset: the corresponding normalized emission spectra at various excitation wavelengths. (**C**) Emission spectra of CDs and of their alkyl-TPP derivatives (λ*_ex_* = 352 nm). (**D**) Normalized UV–Vis spectra of CDs and of their alkyl-TPP derivatives.

The N-doped CDs were water soluble and their very dilute water solutions exhibited strong blue fluorescence upon excitation in the UV region (Figure 2A, Insert). Their excitation spectrum (Figure 2A) exhibited two absorptive peaks: the peak at 240 nm was suggested to result either from n → σ* (C-OH) transitions [43,55,56,57] or from π → π* transitions of the sp^2^ domains of the carbon network [44,58], while the peak at 352 nm originated from n → π* (C=O and C=N) transitions of the sp^3^-hybridized matrix of oxygen- and nitrogen-containing functional groups [43,55,56,57]. The excitation of the aqueous solution at wavelengths from 320 to 400 nm resulted in nearly excitation-independent emission spectra that were all centered at 460 nm (Figure 2B). Their fluorescence intensity reached a maximum at λ_ex_ = 352 nm and thereafter decreased upon increasing the excitation wavelength. Excitation at wavelengths above 400 nm resulted in a shift in the emission maximum to ca. 470 nm, albeit with very low intensity. The fluorescence intensity, as a function of pH, was stable within the pH range 4−10 but decreased both at lower and higher pH values (Figure S6, Supplementary Material). This effect can be attributed to the protonation/deprotonation of the carboxylic or amino groups located at the surfaces of carbon dots (at low pH values, the carboxylic groups become neutral and the amino groups become fully protonated, while the opposite takes place at high pH values), which in turn affect the electronic states of the emission centers.

Moreover, as observed with CDs of the same origin [1,2,7,34], their fluorescence intensity is quenched by metal ions. Indeed, as shown in Figure S7, Supplementary Material, Fe(III) ions strongly decreased their fluorescence intensity, while Fe(II) was a much less effective quencher. The corresponding values of the apparent Stern–Volmer quenching constants, K_SV_, were 5.6 × 10^3^ M^−1^ and 3.8 × 10^2^ M^−1^ for Fe(III) and Fe(II), respectively. The quenching followed the Stern–Volmer law, suggesting the process to be a bimolecular interaction and that fluorescence is, primarily, produced by one emission center; indeed, if the fluorescence is produced by more than one excited center, the quenching constants for the various states would differ and deviation from this law should be observed [59]. Moreover, at least for Fe(III), the K_SV_ value of 5.6 × 10^3^ M^−1^ was two to three orders of magnitude larger than would be observed in water for diffusion-limited quenching, suggesting that these ions were most probably bound to CDs [60]. The fluorescence spectra of the functionalized CDs after excitation at 352 nm were almost identical to the original CD spectrum (Figure 2C) and their corresponding QY was 44.6% and 46.5% for the C_4_TPP or C_10_TPP, respectively. The slightly reduced values compared to the CDs (48.5%) could be attributed to the low absorbance of the TPP moieties in the excitation wavelength (see below). The corresponding emission spectra of the rhodamine-tagged CD•Rh, CD-C_4_TPP•Rh or CD-C_10_TPP•Rh after excitation at 352 nm had the same characteristic profile, with the addition of a low-intensity shoulder at 580 nm (Figure S8, Supplementary Material). Rh had a weak absorption peak at 352 nm, as was evident in the respective excitation spectrum or in the absorption spectrum of Rh (Figure S9, Supplementary Material). Therefore, this weak band originated from the presence of Rh in the carbon dots and was not a result of a possible energy transfer effect between the active centers of CDs and Rh. Further excitation of rhodamine-tagged carbon dots at 558 nm yielded the fluorescence spectra of rhodamine (Figure S8, Supplementary Material).

The absorption spectrum of CDs (Figure 2D) displayed one band centered at 352 nm and a broad one at approximately 250 nm. In accordance with their excitation spectrum, these bands could be ascribed to the n → π* and n → σ* transitions, respectively [43,55,56,57]. In the corresponding absorption spectra of CD-C_4_TPP or CD-C_10_TPP (Figure 2D), the band at 352 nm as well as the bands of the TPP moieties at 274 and 266 nm were also present [61,62] (for comparison, the UV–Vis spectra of (4-carboxybutyl)triphenylphosphonium bromide and of (10-carboxydecyl) triphenylphosphonium bromide are shown in Figure S10, Supplementary Material). The absorption spectra of the rhodamine derivatives CD•Rh, CD-C_4_TPP•Rh or CD-C_10_TPP•Rh in water were the result of simple addition of the corresponding spectra featuring the rhodamine band centered at 558 nm and the bands of CD and of the TPP moieties (Figure S9, Supplementary Material) without any additional features.

### 2.3. Cancer Cell Uptake and Toxicity Profiles of CD Derivatives

**Figure 3:**
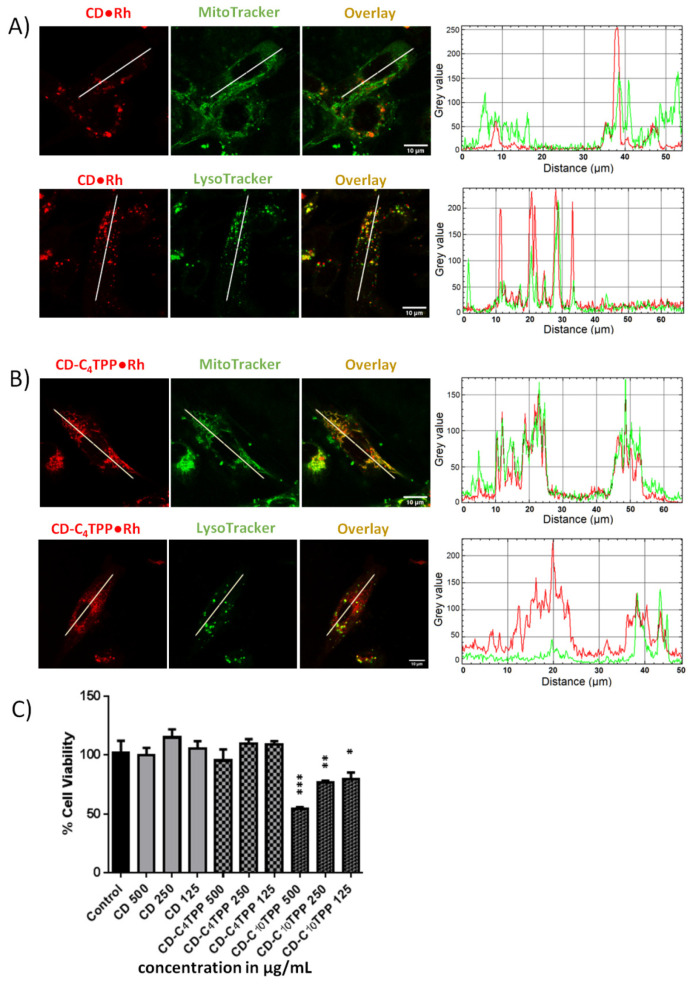
CD•Rh and CD-C4TPP•Rh internalization in live MDA-MB-231 cells. (**A**,**B**) Cells were treated with 25 μg/mL of CD•Rh and CD-C4TPP•Rh for 24 h followed by treatment for 15 min with 100 nM of MitoTracker Green FM and LysoTracker Green DND-26. Overlay depicts co-localization of green and red fluorescence. Plots depict fluorescence intensity profiles along straight white lines. Pearson’s correlation coefficient for CD•Rh was calculated at 0.36 for MitoTracker and 0.63 for LysoTracker (**A**), whereas for CD-C4TPP•Rh at 0.68 for MitoTracker and 0.57 for Lysotracker (**B**); scale bar: 10 μm. (**C**) Cytotoxicity of CD and its derivatives in MDA-MB-231 cells. Cells were treated with increasing concentrations (125–500 μg/mL) of CD, CD-C4TPP and CD-C10TPP for 24 h. Cell viability was assessed by the MTT assay. Results are expressed as the mean ± standard error for at least three independent experiments and were analyzed using the Student *t*-test. Significance is relative to respective controls. * *p* > 0.05, ** *p* > 0.01, *** *p* > 0.001.

In order to evaluate CDs’ suitability for bioimaging applications, we initially performed cytotoxicity tests (MTT) to establish safe treatment concentrations. Only derivatives with a medium degree of functionalization were selected (~50%), given that higher functionalization (>50%) results in reduced water solubility and significant cytotoxicity (Figure S11, Supplementary Material). After a 24 h treatment with increasing concentrations (125–500 μg/mL) of CD, CD-C_4_TPP and CD-C_10_TPP in MDA-MB-231 cancer cells, it was found that CD and CD-C_4_TPP did not result in any toxicity even at high concentrations, whereas CD-C_10_TPP was found to be toxic in a concentration-dependent manner, compared to the control (Figure 3C). The observed cytotoxicity is in line with previous studies that highlighted the toxicity of lipophilic TPP derivatives, especially those with single long aliphatic chains [63,64,65]. It was proposed that the possible mechanism related to the observed toxicity is the increased accumulation of the lipophilic TPP moieties in the mitochondrial membrane, which confers its destabilization, finally leading to membrane depolarization, mitochondrial membrane potential loss or even disruption of respiration and of ATP synthesis. The tendency for membrane accumulation of these specific derivatives can be rationalized by the structural similarity between the long-alkyl-chain TPP derivatives and lysophospholipids; the latter are well known to be cytotoxic by preferentially accumulating and destabilizing biomembranes [63].

Upon establishing non-toxic concentrations, we performed confocal microscopy to evaluate CDs’ cell uptake ability. Cells were treated for 24 h with 25 μg/mL of all rhodamine B-functionalized derivatives and were subsequently co-stained with MitoTracker Green and LysoTracker Green, to evaluate possible co-localization and, therefore, their cellular compartmentalization. Our data demonstrate successful cell uptake and efficient detection of all three derivatives; however, CD-C_10_TPP•Rh was found to disrupt cell integrity even at low concentrations, thus preventing us from drawing conclusive results (data not shown). Upon analysis with Pearson’s correlation coefficient (PCC) for the red and green channels of each multichannel image, and also by plotting the fluorescence intensity profiles on a particular straight line, we observed that CD•Rh was mostly located in lysosomes and far less in mitochondria, with PCC of 0.63 for LysoTracker and 0.36 for MitoTracker (Figure 3A). On the contrary, CD-C_4_TPP•Rh was located both in mitochondria and lysosomes, with PCC 0.68 for MitoTracker and 0.57 for Lysotracker (Figure 3B).

**Figure 4:**
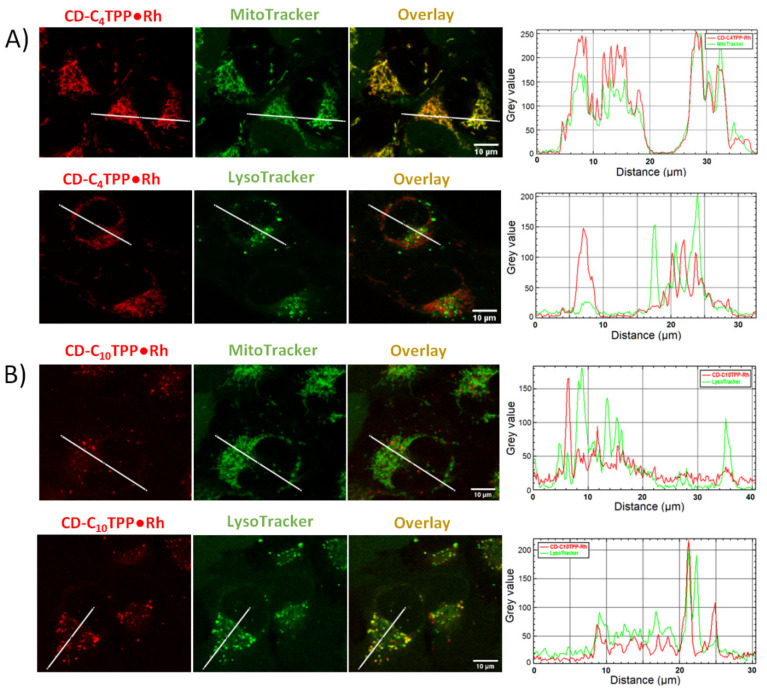
CD-C4TPP•Rh and CD-C10TPP•Rh internalization in live MDA-MB-231 cells. (**A**,**B**) Cells were treated with 50 μg/mL of CD-C4TPP•Rh and CD-C10TPP•Rh for 3 h followed by treatment for 15 min with 100 nM of MitoTracker GreenFM and LysoTracker Green DND-26. Overlay depicts co-localization of green and red fluorescence. Plots depict fluorescence intensity profiles along straight white lines. Pearson’s correlation coefficient for CD-C4TPP•Rh was calculated at 0.85 for MitoTracker and 0.51 for LysoTracker (**A**), whereas for CD-C10TPP•Rh at 0.41 for MitoTracker and 0.63 for LysoTracker (**B**); scale bar: 10 μm.

Having demonstrated that TPP drives, although not exclusively, the C_4_TPP functionalized CDs into mitochondria, we repeated the confocal microscopy experiments to investigate whether mitochondrial localization is time-dependent. Cells were treated with a slightly higher concentration of 50 μg/mL of CD-C_4_TPP•Rh and CD-C_10_•TPP-Rh for 3 h and co-localization analysis was performed as aforementioned. Our data demonstrate a much higher localization of CD-C_4_TPP•Rh to mitochondria within 3 h compared to 24 h incubation time, as PCC was calculated at 0.85 for MitoTracker and at 0.51 for LysoTracker (Figure 4A), while CD-C_10_TPP•Rh, which is not toxic under these conditions but is preferably localized in lysosomes rather than in mitochondria, had a PCC of 0.41 for MitoTracker and PCC 0.63 for LysoTracker (Figure 4B).

### 2.4. Cancer Cell Internalization Properties of CD Derivatives

**Figure 5:**
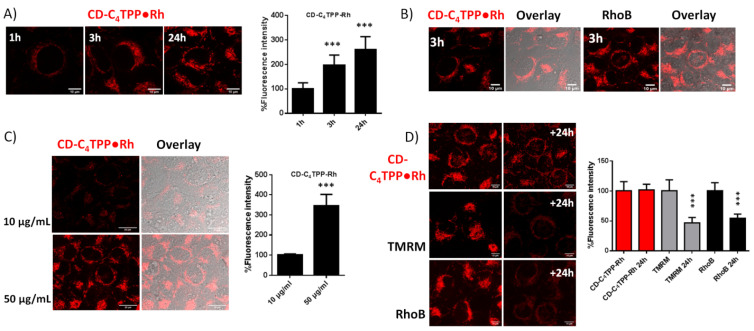
Internalization and retention patterns of CD-C4TPP•Rh in live MDA-MB-231 cells. Cells were treated with: (**A**) CD-C4TPP•Rh 50 μg/mL for 1, 3 and 24 h, (**B**) 50 μg/mL CD-C4TPP•Rh (RhoB 350 nM) and with plain RhoB (350 nM) for 3 h, (**C**) 50 and 10 μg/mL CD-C4TPP•Rh for 24 h, (**D**) 50 μg/mL CD-C4TPP•Rh for 3 h, 20 nM TMRM and 700 nM RhoB for 30 min. Following the above-mentioned incubation times, the compounds were then removed and cells were left in culture for 24 h. Respective graphs depict % quantification of fluorescence intensities. Overlay depicts bright field and red channel. Scale bar: 10 and 20 μm. Results are expressed as the mean ± standard error for at least three independent experiments and were analyzed using the Student *t*-test. Significance is relative to respective controls. *** *p* > 0.001.

Our confocal microscopy data demonstrated promising internalization ability and detection with selectivity to mitochondria for the derivative CD-C_4_TPP•Rh, at low concentrations and at different incubation periods. Therefore, we proceeded with additional confocal experiments with this particular derivative, employing various concentrations and incubation time points. Total fluorescent intensity analysis showed that the internalization of CD-C_4_TPP•Rh was almost proportional to its concentration (Figure 5C), while its accumulation was time-dependent (Figure 5A). Notably, CD-C_4_TPP•Rh fluorescent detection was possible even at concentrations as low as 10 μg/mL (Figure 5A).

To evaluate its potency as a mitochondrial marker, we further studied its internalization properties with respect to known mitochondrial probes, such as tetramethylrhodamine (TMRM) and rhodamine B (RhodB). Initially, we performed 3 h of treatment with 50 μg/mL of CD-C_4_TPP•Rh and 350 nM of Rhodamine B, which is the equivalent concentration of Rh in CD-C_4_TPP•Rh, and observed similar cell internalization patterns, as expected (Figure 5B). Next, we compared CD-C_4_TPP•Rh retention properties against TMRM and RhodB in comparable treatment concentrations after 24 h, following an initial 3 h incubation treatment and subsequent removal of compounds. Remarkably, total fluorescent intensity analysis demonstrated that its red fluorescent signal was detected almost unaltered, thus proving that CD-C_4_TPP•Rh was retained in the cell after 24 h without resulting in cell toxicity (Figure 5C), compared to the significant loss of the fluorescent signal of plain RhodB or TMRM, indicating their efflux from the cell (Figure 5D).

**Figure 6:**
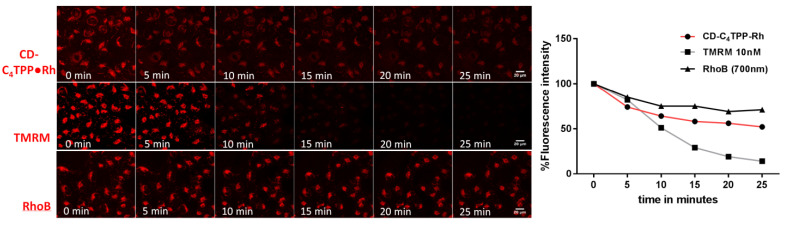
Photostability of CD-C4TPP•Rh vs. TMRM and RhoB in live MDA-MB-231 cells and the corresponding fluorescence quantification (%) over time. Cells were treated with 50 μg/mL CD-C4TPP•Rh for 3 h, and TMRM 10 nM or RhoB 700 nM for 30 min. Photostability was assessed under continuous laser irradiation with 5 min intervals. Scale bar: 20 μm.

Due to low photostability and long-term toxicity, the use of commercial mitochondrial probes in the process of long-term tracking is often limited. Hence, we further tested CD-C_4_TPP•Rh’s photostability in comparison to RhodB and TMRM by measuring its fluorescence intensity in MDA-MB-231 cells under continuous laser irradiation for 25 min. Our results showed that, after 25 min, there was a ~48% drop in CD-C_4_TPP•Rh’s fluorescence intensity, similar to RhoB’s fluorescent pattern, whereas TMRM’s fluorescence intensity declined by 86% (Figure 6). Overall, we conclude that CD-C_4_TPP•Rh is photostable, retained well and is safe and suitable for long-term cell imaging.

### 2.5. CD-C_4_TPP•Rh Selective Mitochondrial Staining and Internalization Mechanism in MDA-MB-293 Cells

**Figure 7:**
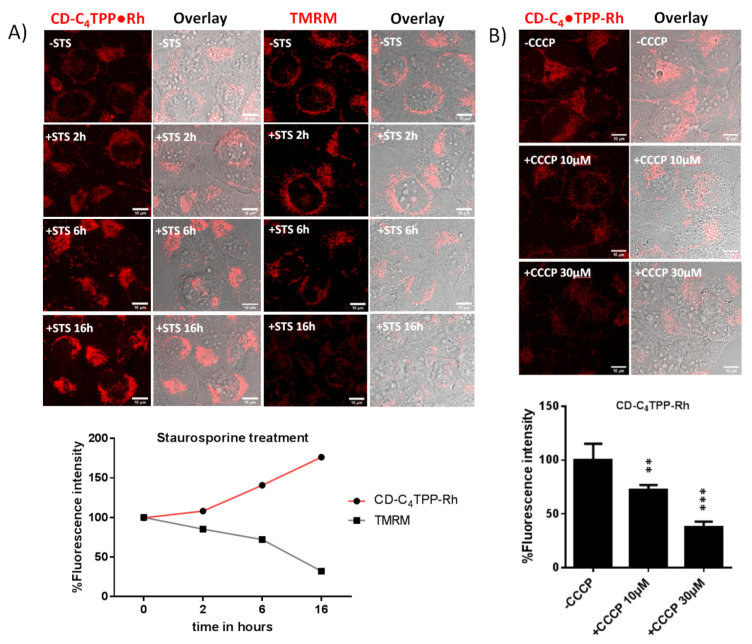
Staining patterns of CD-C4TPP•Rh in live MDA-MB-231 cells under various treatments and the corresponding fluorescence quantification over time. (**A**) Cells were treated with CD-C4TPP•Rh 50 μg/mL for 3 h, and TMRM 10 nM for 30 min following treatment with 2 μM of staurosporine (STS) for 2, 6 and 16 h. The respective graph depicts % quantification of fluorescence intensities over time. (**B**) Cells were treated with CD-C4TPP•Rh 50 μg/mL for 3 h following treatment with 10 and 30 μM of CCCP for 30 min. Respective graphs depict % quantification of fluorescence intensities. Overlay depicts bright field and red channel. Scale bar: 10 μm. Results are expressed as the mean ± standard error for at least three independent experiments and were analyzed using the Student *t*-test. Significance is relative to respective controls. ** *p* > 0.01, *** *p* > 0.001.

To establish whether CD-C_4_TPP•Rh internalization is affected by alterations in cells’ normal function, we pretreated cells with two different stress agents, one resulting in apoptosis and the other resulting in membrane potential depolarization. First, we pre-treated MDA-MB-293 cells with staurosporine (STS), a prototypical ATP-competitive kinase inhibitor and a known pro-apoptotic agent, for 2, 6 and 16 h prior to treating cells with 50 μg/mL of CD-C_4_TPP•Rh for 3 h. For comparison purposes, cells were also treated with TMRM. Our results showed that, although the fluorescence of TMRM was diminished after 2 h of STS treatment in a time-dependent manner, the fluorescence of CD-C_4_TPP•Rh was increased inversely (Figure 7A). This suggests that CD-C_4_TPP•Rh can potentially be used as an early apoptotic marker.

Secondly, we treated cells with carbonyl cyanide 3-chlorophenylhydrazone (CCCP), an uncoupler agent that can decrease the mitochondrial membrane potential (Δψ_m_), to investigate whether CD-C_4_TPP•Rh internalization is dependent on changes in Δψ_m_. Upon treatment with increasing concentrations of CCCP, we observed a dramatic drop in CD-C_4_TPP•Rh’s fluorescence, proving that its uptake is influenced by major Δψ_m_ alterations (Figure 7B).

### 2.6. CD-C_4_TPP•Rh Cancer Cell Targeting Ability

**Figure 8:**
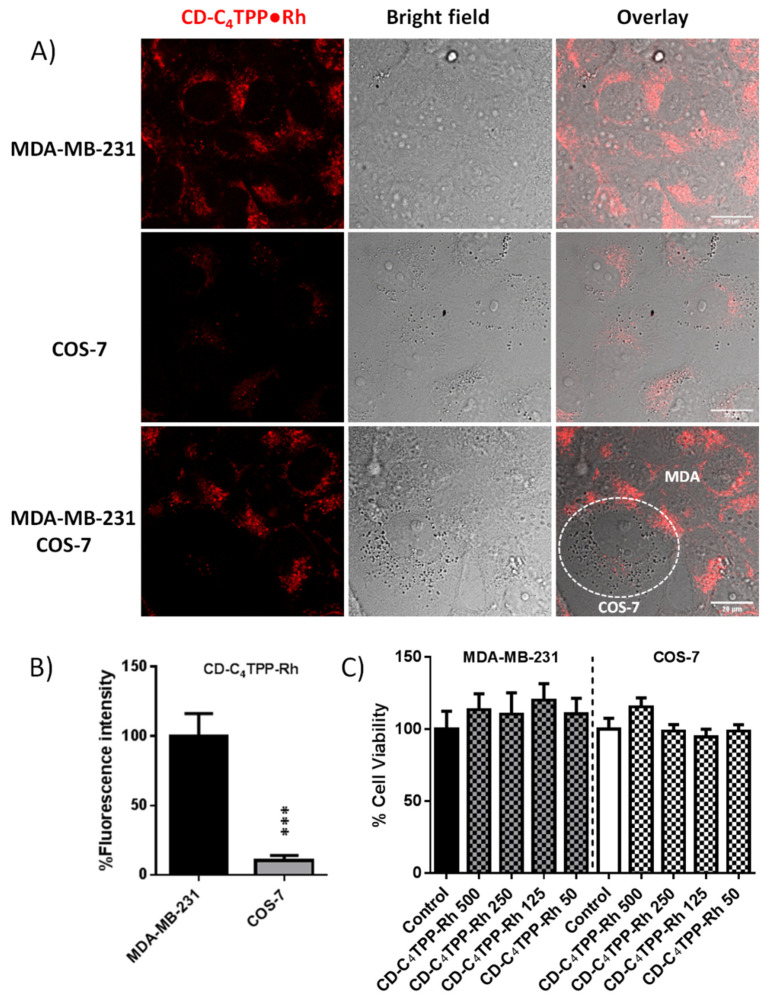
(**A**) Comparison of CD-C4TPP•Rh internalization in live COS-7 and MDA-MB-231 cells, as well as in co-culture of both cell lines. Cells were treated with 50 μg/mL of CD-C4TPP•Rh for 24 h. Overlay depicts bright field and red channel. Scale bar: 20 μm. (**B**) Fluorescence quantification (%) of different internalization intensities between cell lines. (**C**) Cytotoxicity of CD-C4TPP•Rh in MDA-MB-231 cells and COS-7 cells. Cells were treated with increasing concentrations (50–500 μg/mL) of CD-C4TPP•Rh for 24 h. Cell viability was assessed by the MTT assay. Results are expressed as the mean ± standard error for at least three independent experiments and were analyzed using the Student *t*-test. Significance is relative to respective controls. *** *p* > 0.001.

Having established CD-C_4_TPP•Rh’s excellent mitochondrial targeting and cell compatibility, we further investigated whether its cell uptake differentiates based on cell status. For this reason, we repeated the confocal experiments individually in cancerous MDA-MB-231 and normal COS-7 cells to search for differences in CD-C_4_TPP•Rh internalization. Upon ensuring the lack of cytotoxicity of CD-C_4_TPP•Rh in both cell lines after 24 h (Figure 8C), we proceeded in treating them with 50 μg/mL CD-C_4_TPP•Rh. We observed a striking difference in the cell uptake ratio between the non-cancerous and cancerous cells, as CD-C_4_TPP•Rh internalization was much lower in COS-7 compared to MDA-MB-231 cells, as demonstrated by the lack of red fluorescence (Figure 8A,B). To further validate our observation, we co-cultured both cell lines under the same conditions, reaching the same conclusion and confirming that CD-C_4_•TPP is a promising candidate for the selective imaging of cancerous cells (Figure 8A).

## 3. Discussion

It is currently widely accepted that the fluorescence properties of CDs are significantly enhanced and modulated upon surface passivation with heteroatoms [30,66]. In particular, surface passivation (doping) with nitrogen is one of the most preferred strategies, due to the comparable atomic size of nitrogen with carbon and the presence of five valence electrons for bonding to carbon [30]. Additional advantages are the low cost of starting materials—in most cases, an organic acid and an amine—the ease of production and the high quantum yield attainable, if the reaction conditions are carefully chosen. Thus, citric acid-based CDs were prepared through bottom-up polymerization–cyclization–pyrolysis of citric acid and amine-containing compounds exhibiting high quantum yields and excitation-independent fluorescence emission [34,42,43,44,45,46,67,68,69], while they are also very well characterized regarding the variety of their emission centers [1,42,43,47,70]. Our synthetic approach was to determine the irradiation timeframe for the specific microwave set-up that would produce the highest QY, and then to fine-tune the molar ratio for achieving the best result. We were thus able to obtain CDs with a QY of nearly 50%, i.e., close to the currently available standard, quinine sulfate (QY = 0.577). Our findings that increased reaction times are detrimental for the QY of the products and that the optimum molar ratio of the reactants is close to 1:1 are in line with previous literature data [47,71]. The electronic and optical properties, NMR studies, as well as the TEM images and the X-ray diffractogram point to sp^2^ crystalline graphitic carbon cores together with amorphous/disordered graphitic sp^2^ structures and sp^3^-hybridized domains containing oxygen and nitrogen. The quenching of fluorescence, following strictly the Stern–Volmer law, suggests that the fluorescence properties observed are—primarily—due to one highly fluorescent emission center, in line with previous reports pointing to certain small fluorophores as the most prevalent emission centers of N-doped carbon dots [45,46,67,68,69]. Overall, in every respect, the spectra are in line with what is commonly observed in CDs obtained through carbonization of citric acid with amines [42,45,46,53,55,67].

Although N-doped CDs exhibit strong optical absorption in the UV region and are highly fluorescent when excited in this spectral range, they possess inefficient absorption in the visible wavelength region, limiting their use in cell or in vivo imaging applications. In addition, both quenching effects as well as the autofluorescence from cells in the UV or short-wavelength visible region limit their application or necessitate their use at high concentrations. Therefore, research is now focusing on synthesizing novel, but more complicated, CD structures that fluoresce brightly when excited in the visible wavelength region—particularly in the red region—to be applied in bioimaging [2,37,38,72]. However, the quantum yield in these cases is often lower than that of typical organic fluorescent tags employed in bioimaging [2,73,74,75]. In this study, we utilize the low-cost, easily synthesized and benign N-doped carbon dots for their functionalization with rhodamine. Thus, we obtain tagged CDs with high quantum yield in the UV region and the efficient fluorescence properties of the rhodamine moiety in the visible region. The amount employed (~0.4% *w/w*) proved very sufficient for confocal microscopy cell imaging, while it is low enough not to pose any concerns regarding possible detrimental effects on the physicochemical and biological properties of CDs.

Initial experiments employing the non-rhodamine-functionalized CD derivatives, i.e., CD, CD-C_4_TPP, CD-C_10_TPP, in confocal microscopy cell imaging dictated the use of high concentrations of CDs (250–500 μg/mL) in order to attain a sufficient fluorescent signal in the blue channel. This resulted in inconclusive results concerning their subcellular localization, as the fluorescent signals were found throughout the cell interior. Since there is a certain mass and, therefore, limited capacity of subcellular organelles, such as mitochondria or lysosomes, to internalize these nanoparticles, the selective accumulation in specific sites when high concentrations are used is doubtful, even if the nanoparticles impose no stress on the cells, which is also to be considered. Rhodamine functionalization, on the other hand, increases the imaging options and allows the use of considerably lower concentrations (10 μg/mL).

Consequently, the N-doped CDs as well as the two alkyl-TPP functionalized CD derivatives that were conjugated with rhodamine B (CD-Rh, CD-C_4_TPP•Rh, CD-C_10_TPP•Rh) were in vitro evaluated for their cellular compatibility, mitochondrial selectivity and fluorescent properties. Out of the three, plain CD and TPP-functionalized CD-C_4_TPP•Rh were found to be the safest compounds, causing no cell toxicity after 24 h, even up to high concentrations of 500 μg/mL. Moreover, all derivatives were successfully internalized in cancer cells yet differently distributed, as CD-Rh was mostly entrapped in lysosomes, compared to TPP-functionalized CD-C_4_TPP•Rh, which was primarily located in mitochondria. Our data are consistent with the literature, in which N-doped carbon dots are mainly located in acidic lysosomes [22], whereas CDs functionalized with cationic mitochondrial moieties, such as TPP, are being driven to mitochondria [76]. Interestingly, however, CD-C_10_TPP•Rh was found to preferably locate in lysosomes despite the presence of alkyl-TPP moieties.

Lysosomes prevent the subcellular distribution of various chemical entities by degrading them. The majority of clinically approved drugs, which are both weak bases and lipophilic in nature, accumulate preferably in the acidic intracellular compartments of lysosomes, since the lysosomal pH is acidic (pH ≈ 5). This phenomenon, referred to as “acid trapping” or “lysosomotropism”, can lead to the lysosomal accumulation of such compounds at concentrations up to 100-1000-fold higher than extracellular concentrations [29]. On the other hand, the pH in the interior of mitochondria is alkaline (pH ≈ 8.0), which suggests that weak bases are excluded from entering their interior. Mathematical models and computational approaches that describe and rationalize the transport and distribution at cellular and subcellular levels of small molecules predict that polar weak bases preferably accumulate in lysosomes [77]. Accumulation in mitochondria of cationic compounds will only be favored if the permeability of their ionic and neutral forms is roughly equal—as is the case for delocalized cations such as TPP—and also if they are only moderately lipophilic [77,78]. It is, therefore, clear that mitochondrial localization is affected not only by the presence of delocalized lipophilic cations but also by the overall basicity and lipophilicity of the compounds. While our TPP-functionalized CDs have the same basic character, the lipophilicity of the C_10_TPP•Rh is higher and this apparently results in lysosomal localization for this derivative.

Focusing on CD-C_4_TPP•Rh, we next investigated whether this accumulation is time-dependent, given that mitochondrial capacity is limited. A shorter incubation period of 3 h resulted in much higher internalization and specificity of CD-C_4_TPP•Rh to mitochondria, which is in accordance with another recent study, where the optimal concentration of CDs was also found to be 50 μg/mL for 4 h incubation [37]. Although CD-C_10_TPP•Rh was proven to be safe at low concentrations, it nevertheless failed in analogous accumulation, as it was mostly located in lysosomes, as explicated above. Furthermore, optimization of CD-C_4_TPP•Rh’s mitochondrial staining ability was performed at different time points and concentrations. It was found that it was detected inside the cell in a time- and concentration-dependent manner, even at very low concentrations (10 μg/mL), and as quickly as within 1 h of incubation. In another study, successful cellular uptake of CDs was also demonstrated; however, a significantly higher concentration (100 μg/mL) of CDs was required in order to be detected in less than 1 h, relying on CDs’ inherent fluorescent properties [72]. Moreover, our data demonstrated that CD-C_4_TPP•Rh was retained stably within the cell compared to known mitochondrial staining probes, such as TMRM and plain RhodB, which were no longer detected inside the cell after 24 h of culture. These results are consistent with literature data highlighting P-glycoprotein and the multidrug resistance protein-mediated efflux of rhodamine dyes in resistant cells [79]. Since photostability is a crucial factor for fluorescent probes, we also compared CD-C_4_TPP•Rh’s performance upon continuous laser irradiation against TMRM, with RhodB as a positive control. Our results clearly show that CD-C_4_TPP•Rh outperforms TMRM significantly, indicating great potential for long-lasting mitochondrial tracking. Given that our experiments were performed in cancer cells, we expanded our search on whether CD-C_4_TPP•Rh can track mitochondria universally, regardless of cell type and background. Interestingly, CD-C_4_TPP•Rh was selectively accumulated in cancer cells and not in normal cells, demonstrating prominent cell differentiation ability based on cell malignancy, possibly due to the different Δψ_m_ status between normal and cancer cells. Cancer cells have generally more negative Δψ_m_ compared to normal cells [80], and a more hyperpolarized Δψ_m_ is associated with the aggressiveness of cancer cells, such as in the triple-negative MDA-MB-231 cancer cells used herein [81], in stark contrast to COS-7 cells or to MDA-MB-231 cells treated with CCCP, an agent that is known to decrease Δψ_m_. To further validate our assumption and study CD-C_4_TPP•Rh’s internalization mechanism, we treated MDA-MB-231 with two different stressor agents, CCCP and staurosporine. In the first case, treatment with increasing concentrations of CCCP, an agent known to quickly depolarize mitochondrial membrane potential [82], triggering the collapse of the mitochondrial structure [83], led to a proportional decrease in CD-C_4_TPP•Rh cell uptake, thus indicating that CD-C_4_TPP•Rh internalization is indeed Δψ_m_-dependent. In the second case, time-dependent treatment with pro-apoptotic agent staurosporine lead to an increase in the cell uptake of CD-C_4_TPP•Rh with a concomitant decrease in TMRM uptake, which is in line with a similar study where an inverse TMRM and CD fluorescent pattern after 6 h of staurosporine treatment was reported [72]. Apoptosis is a slow process, during which mitochondria undergo an initial hyperpolarization phase, followed by swelling, Δψ_m_ dissipation, cytochrome c release and, finally, cell death [84]. Hence, we deduce that that CD-C_4_TPP•Rh are being retained in mitochondria and are slowly released into the cytoplasm in the final phase of apoptosis, in contrast to the fast efflux of TMRM from the cell, indicating that perhaps they could be used as an apoptotic indicator.

## 4. Materials and Methods

### 4.1. Chemicals and Reagents

Citric acid (99.8%), ethylenediamine (≥99%), *N*,*N*’-dicyclohexylcarbodiimide (DCC, 99%*), N*-hydroxysuccinimide (NHS, 98%), triethylamine (TEA, ≥99%), triphenylphosphine (98%), 11-bromoundecanoic acid (≥99%), (4-carboxybutyl)triphenylphosphonium bromide (98%), ninhydrin (≥98%) and rhodamine B isothiocyanate were purchased from Sigma-Aldrich Ltd. (Poole, UK). Quinine sulfate (99+%) was supplied by Janssen Chimica. Cell culture RPMI 1640 medium with stable glutamine, fetal bovine serum (FBS), penicillin/streptomycin, phosphate-buffered saline (PBS), trypsin/EDTA, MitoTracker GreenFM, LysoTracker Green DND-26, tetramethyl rhodamine methyl ester (TMRM), carbonyl cyanide m-chlorophenylhydrazone (CCCP, 98%) and staurosporine (STS, >98%) were all purchased from ThermoFisher Scientific (Waltham, VA, USA). Thiazolyl blue tetrazolium bromide (MTT, 98%) was purchased from Merck KGaA (Calbiochem, Darmstadt, Germany). All other reagents and solvents were of analytical grade and used without further purification.

### 4.2. Synthesis of Multifunctional CDs

#### 4.2.1. Synthesis of N-Doped CDs

To citric acid (CA, 1 g, 5.21 mmol) dissolved in 10 mL doubly distilled water, various quantities of ethylenediamine (EDA, 4.20 to 6.29 mmol) were added and the resulting solutions were heated in a microwave oven (800 W) for different time periods (1–4 min) in order to find the optimum conditions for maximum quantum yield (QY). The resulting products were dissolved in 10 mL of water, filtered through a 0.22 μm syringe filter and extensively dialyzed against doubly distilled water, employing a dialysis membrane of MW 12,000 g mol^−1^ cut-off, until the outer solution was non-fluorescent. Following lyophilization, the QY of the samples was evaluated. Samples obtained employing a molar ratio of CA:EDA = 1:1 and microwave-irradiated for different time periods (1.5 to 4.0 min) indicated that the optimum irradiation time that resulted in CDs having the highest QY was 2 min (Table S1, Supplementary Material); samples prepared employing various CA:EDA molar ratios and irradiated for 2 min showed that the highest QY value was obtained at 0.90:1 molar ratio (Table S2, Supplementary Material). The mass percentage yield for this preparation, after the thorough purification steps, was 12%. Therefore, the CDs with QY of 48.5% obtained following the optimum reaction conditions (molar ratio of CA:EDA = 0.90:1.00, irradiation time = 2 min) were used in all subsequent experiments. Their chemical structure was studied by ^1^H- and ^13^C-NMR spectroscopy (Figure S1, Supplementary Material), while the quantification of the amino groups of CDs was carried out by employing the ninhydrin method (see Section 2.3).

^1^H-NMR (500 MHz, D_2_O), *δ* (ppm): 5.5–6.0 (-CH=CH-), 2.2–4.3 (-CH_2_-CH_2_-, CH_2_-N, CH_2_-NHCO, CH_2_-O, CH_2_-CO).

^13^C-NMR (125.1 MHz, D_2_O), *δ* (ppm): 176–181 (COO), 171–173 (NHCO), 150–170 (-CH=CH-), 74 and 72.5 (CH_2_-O), 35–45 (-CH_2_-CH_2_-, CH_2_-NHCO, CH_2_-CO).

#### 4.2.2. Synthesis TPP-Functionalized CDs

The functionalization of CDs with alkylated triphenylphosphonium functional groups with either butyl or decyl alkyl chains—see Scheme 1—was performed following an analogous synthetic route, as described in detail in our previous publications [40,41]. In short, initially, the synthesis of (10-carboxydecyl)triphenylphosphonium bromide was achieved by the reaction of triphenylphosphine with 11-bromoundecanoic acid in anhydrous DMF under an inert atmosphere. The pure product was obtained after recrystallization in ethyl acetate. For the preparation of butyl-triphenylphosphonium-functionalized CDs (CD-C_4_TPP) or decyl-triphenylphosphonium-functionalized CDs (CD-C_10_TPP), various quantities (10 to 25 mmol) of either (4-carboxybutyl)triphenylphosphonium bromide or (10-carboxydecyl)triphenylphosphonium bromide were dissolved in dry DMSO, and a 30% molar excess of DCC and TEA also dissolved in DMSO was added (final volume 5 mL) and left for 1 h, at 25 °C. Subsequently, NHS (30% molar excess) dissolved in DMSO was added and left overnight at 25 °C. The reaction mixture was filtrated and added to a DMSO solution of CDs (100 mg, 4 mL). The reaction was allowed to complete at 25 °C for around 24 h, with constant stirring under an inert atmosphere. The solvent was partially removed under vacuum and the reaction product was twice precipitated in dioxane and finally dried under vacuum. After drying, it was redissolved in water, dialyzed against doubly distilled water for 2 days, employing a dialysis membrane of MW 12,000 g mol^−1^ cut-off, and finally obtained after lyophilization. The various C_4_TPP- or C_10_TPP-functionalized carbon dots synthesized employing carbon dots with different degrees of functionalization are shown in Table S3, Supplementary Material. Their chemical structure was studied by NMR spectroscopy (Figure S2, Supplementary Material). In a typical experiment, where 0.15 mmol of either CD-C_4_TPP or CD-C_10_TPP was interacted with 100 mg of CDs, it was found that, on average, 0.57 mmol decyl-TPP/gr CDs (0.29 g C_10_TPP/g CDs) or 0.51 mmol butyl-TPP (0.21 g C_4_TPP/g CDs) groups were conjugated to CDs, as determined by ^1^H NMR, using maleic acid as an internal standard (the reaction mass percentage yields were 54% and 49% for the CD-C_4_TPP or CD-C_10_TPP, respectively). The quantification of the amino groups of CDs was also carried out by the ninhydrin method (see Section 4.3).

^1^H-NMR for CD-C_4_TPP (500 MHz, D_2_O), *δ* (ppm): 8.0–7.6 (m, aromatic H of TPP), 6.0-5.5 (-CH=CH-), 4.3–2.3 (-CH_2_-CH_2_-, CH_2_-N, CH_2_-NHCO, CH_2_-O, CH_2_-CO), 2.3–2.0 (NHCOCH_2_), 1.7 (CH_2_CH_2_CH_2_P^+^Ph_3_), 1.6 (CH_2_CH_2_P^+^Ph_3_).

^1^H-NMR for CD-C_10_TPP (500 MHz, D_2_O), *δ* (ppm): 8.0–7.6 (m, aromatic H of TPP), 6.0–5.5 (-CH=CH-), 4.3–2.3 (-CH_2_-CH_2_-, CH_2_-N, CH_2_-NHCO, CH_2_-O, CH_2_-CO), 2.3–2.0 (NHCOCH_2_), 1.70–1.55 (CH_2_CH_2_CH_2_P^+^Ph_3_), 1.50–1.26 (m, CH_2_CH_2_P^+^Ph_3_, NHCOCH_2_CH_2_), 1.25–0.85 (m, aliphatic CH_2_).

#### 4.2.3. Synthesis of Rhodamine (Rh)-Functionalized CDs

Rhodamine-labelled CD, CD-C_4_TPP and CD-C_10_TPP were prepared by reacting the corresponding N-doped CDs, CD-C_4_TPP or CD-C_10_TPP (50 mg) with rhodamine B isothiocyanate (5 mg) in dry DMSO in the presence of TEA. The reaction was allowed to complete at 25 °C for approximately 24 h with constant stirring in the dark under an inert atmosphere. The solvent was partially removed under vacuum and the product was twice precipitated in dioxane, filtered and dried under vacuum. After drying, it was redissolved in water and dialyzed against doubly distilled water for 2 days, employing a dialysis membrane of MW 12,000 g mol^−1^ cut-off. The final products CD•Rh, CD-C_4_TPP•Rh or CD-C_10_TPP•Rh were obtained after lyophilization. The amount of rhodamine B (Rh) in all products (~0.4% *w/w*) was determined by dissolving a known amount in water and registering the absorbance at 558 nm. The respective calibration curve of rhodamine B in water was also determined under the same conditions. It was thus found that the concentration of Rh was 7.0 ± 0.2 μM in 1 mg/mL solutions of Rh-labelled carbon dots, which corresponded to 3.75 ± 0.15 μg of Rh per mg of carbon dots.

### 4.3. Characterization Techniques

^1^H and ^13^C NMR spectra were recorded in D_2_O by a Bruker Avance DRX spectrometer operating at 500 and 125.1 MHz, respectively. FTIR studies were performed using a Nicolet 6700 spectrometer (Thermo Scientific, Waltham, MA, USA) equipped with an attenuated total reflectance accessory with a diamond crystal (Smart Orbit, Thermo Electron Corporation, Madison, WI, USA). Samples were firmly pressed against the diamond, and spectra were recorded at 4 cm^−1^ resolution. A minimum of 64 scans were collected and signal averaged. *ζ*-potential measurements were conducted at 22 °C using the ZetaPlus of Brookhaven Instruments Corp. (Long Island, NY, USA) equipped with a 35 mW solid-state laser emitting at 660 nm and with an in-built temperature controller able to stabilize the temperature within ±0.5. From the obtained electrophoretic mobility, the ζ-potential was calculated using Smoluchowski’s equation. Ten measurements were collected for each dispersion and the results were averaged. The standard deviation of the measurements was less than 1%. Dynamic light scattering measurements were performed on an ALV/CGS-3 Compact Goniometer System (ALV GmbH, Germany) using a JDS Uniphase 22 mW He Ne laser operating at 632.8 nm and an Avalanche photodiode detector at an angle of 90°, interfaced with an ALV-5000/EPP multitau digital correlator with 288 channels and an ALV/LSE-5003 light scattering electronics unit for stepper motor drive and limit switch control. For each dispersion, at least ten light scattering measurements were acquired, and the autocorrelation functions were analyzed using the CONTIN algorithm to obtain the apparent hydrodynamic radii distribution. Fits to the correlation functions were made using the software provided by the manufacturer.

Quantification of the primary amino groups of CDs was carried out by their reaction with ninhydrin, which affords the chromophoric compound Ruhemann’s Purple and is extensively used for the detection and the quantitative estimation of the amino groups in a-amino acids, as well as in amine-functionalized nanoparticles [85,86]. Specifically, accurately weighted CDs (3–4 mg) were dissolved in 50 μL of phosphate buffer (pH 7.4, 150 mM), and 500 μL of a 0.35% ninhydrin solution in DMSO was added. The solution was heated in a thermostated water bath at 65 °C for a fixed time of 15 min. The samples were cooled at room temperature, diluted with DMSO (1:100) and their absorbance was registered at 604 nm. Standard alanine solutions were used, following the same procedure as above (final concentration range 1−10 × 10^−5^ M) for the construction of the calibration curve. All measurements were performed in triplicate.

The fluorescence spectra were recorded on a Cary Eclipse fluorescence spectrophotometer from Varian Inc. (Mulgrave, Victoria, Australia). UV–Vis spectra were recorded using a Cary 100 Conc UV−visible spectrophotometer (Varian Inc., Mulgrave, VIC, Australia). The quantum yield (QY) measurements of the CDs were performed by comparison of the wavelength-integrated fluorescence intensity of a number of CD solutions (excited at 352 nm) to those of solutions of the standard quinine sulfate (QY = 0.577, at 0.1 M H_2_SO_4_, 22 °C) [60]. The optical density of the solutions employed was kept below 0.1 at the excitation wavelength, to avoid inner filter effects. The slope (*m*) of the graph of the integrated fluorescence intensity versus the absorbance were determined for both the CDs and the quinine sulfate solutions, and they were used to calculate the QY using the following equation [47]: QY = QY_s_*(*m*/*m*_s_)*(*n*/*n*_s_)^2^, where the subscript “s” refers to the standard and “*n”* refers to the refractive indexes of the media (1.333 and 1.334 are the refractive indexes of distilled water and of 0.1 M H_2_SO_4_ [87], respectively).

Transmission electron microscopy (TEM) experiments were performed by employing a Philips FEI CM20 microscope operating at 200 kV to investigate the size and morphology of the respective nanoparticles. In this case, a droplet of water CD solution was deposited on a carbon-coated 200-mesh copper grid and allowed to evaporate in air. X-ray measurements were performed using a set-up consisting of a Rigaku RUH3R rotating anode generator operating at 50 kV, 100 mA, and producing a beam of λ = 1:5416 Å (K_α_ line of Cu). An R-axis IV double imaging plate (area of 300 × 300 mm^2^) from Rigaku Co., Tokyo, Japan was used as a detector. The pixel size was 100 × 100 μm^2^ and the sample-to-detector distance during experiments was set to 100 mm. Samples were placed in Lindemann capillaries (Hilgenberg Mark tubes of 1 mm inner diameter).

### 4.4. Cell Culture and Treatments

Cells used in this study were the human breast cancer cell lines MDA-MB-231 and the non-cancerous monkey kidney COS-7 (ATCC, Rockville, MD, USA). The cells were grown in RPMI 1640 medium with stable glutamine, supplemented with 10% FBS and 1% penicillin/streptomycin at 37 °C in a 5% CO_2_ humidified atmosphere.

### 4.5. Cytotoxicity Assay

Cells were seeded into 96-well plates at a density of 10 × 10^3^ cells per well. The following day, cells were treated with different concentrations of CD, CD-C_4_TPP, CD-C_10_TPP and CD-C_4_TPP•Rh in complete medium for 24 h, and the mitochondrial redox function of all cell groups was assessed by the MTT assay (Sigma, Neustadt an der Weinstra, Germany). Briefly, cells were incubated with 1 mg/mL MTT-containing medium for 4 h, and following MTT removal, the produced formazan crystals were solubilized in isopropanol. The absorbance was measured with an Infinite M200 plate reader (Tecan Group Ltd., Männedorf, Switzerland) at a wavelength of 570 nm.

### 4.6. Confocal Laser Scanning Microscopy (CLSM) Studies

For all studies described below, cells were seeded on μ–Dish 35 mm dishes (ibidi GmbH, Martinsried, Germany) and left to grow overnight in 2 mL of RPMI medium with 10% FBS under the same conditions as elaborated above in the cell culture section. CLSM studies were performed on an inverted Leica-TCS SP8 confocal microscope (Wetzlar, Germany), with an HC Plan APO 63×, 1.40 N.A. oil immersion objective. A 488 nm laser line from a 25 mW argon ion laser (for MitoTracker and LysoTracker) and a 561 nm laser line (for rhodamine) from a 20 mW DPSS ion laser with high-sensitivity hybrid detectors were used. Images were acquired with the LAS X software (Leica Microsystems CMS GmbH). For comparison purposes, confocal images were acquired using the same settings for all treatment conditions. The total fluorescence intensity was calculated with ImageJ software (http://imagej.net, accessed on 14 July 2021). Briefly, the acquired images were quantified by measuring the integrated density of each image of the red channel corrected by the number of cells per frame. An appropriate threshold was applied to all conditions.

### 4.7. Subcellular Localization of CDs

MDA-MB-231 cells were incubated with 25 and 50 μg/mL of CD•Rh, CD-C_4_TPP•Rh and CD-C_10_TPP•Rh for 3 or 24 h. MitoTracker GreenFM or LysoTracker Green DND-26 were added at a final concentration of 100 nM for 15 min. Co-localization was quantitated using Pearson’s correlation coefficient (PCC) via the Coloc 2 plugin of the Fiji distribution of ImageJ software (http://imagej.net/Coloc_2, accessed on 14 July 2021). Briefly, PCC is a statistic that measures the pixel-by-pixel covariance in the signal levels of two images. Because it subtracts the mean intensity from each pixel’s intensity value, PCC is independent of signal levels and signal offset (background) and can be measured in two-color images without any form of preprocessing [88].

### 4.8. Internalization Properties and Photostability

MDA-MB-231 cells were incubated with 10 or 50 μg/mL of CD-C_4_TPP•Rh for 1, 3 or 24 h. To compare the cell internalization and retention properties of CD-C_4_TPP•Rh, treatment with the known mitochondrial marker tetramethylrhodamine (TMRM) and with rhodamine B (RhoB) was also performed. To assess the photostability of CD-C_4_TPP•Rh, cells were treated with CD-C_4_TPP•Rh, TMRM and RhoB and were subjected to continuous laser irradiation at 561 nm for 25 min. Images were captured ever 5 min.

### 4.9. Differentiation between Cancer and Normal Cells

The cancer cell line MDA-MB-231 and the normal cell line COS-7 were cultured separately and as co-culture in a 1:1 ratio. All cell cultures were treated with the same concentration of CD-C_4_TPP•Rh (50 μg/mL) in order to evaluate its selectivity between these two cell lines.

### 4.10. Selective Mitochondrial Staining under Treatments

To assess the selective mitochondrial staining of CD-C_4_TPP•Rh upon mitochondrial membrane potential alterations and apoptosis induction, cells were treated with carbonyl cyanide 3-chlorophenylhydrazone (CCCP), a known uncoupler agent, and staurosporine, an apoptotic agent. MDA-MB-231 cells were treated with 10 and 30 μΜ of CCCP for 30 min or with 2 μΜ of staurosporine for 2, 6 and 16 h and subsequently with 50 μg/mL of CD-C_4_TPP•Rh for 3 h. A comparison was performed using TMRM, a cell-permeant dye that accumulates in active mitochondria with intact membrane potential, and the red fluorescence signals were measured as aforementioned.

### 4.11. Statistical Analysis

Data are presented as mean ± standard deviation. All experiments were performed independently at least three times. Significance was defined as *p* < 0.05 by using the Student *t*-test.

## 5. Conclusions

Functionalization of N-doped carbon dots with alkyl-TPP moieties does not by itself guarantee mitochondrial localization. The careful selection of the alkyl chain length, functionalization degree and concentration is needed in order to achieve mitotropism and preserve the minimal toxicity and biocompatibility of carbon dots. Conjugation with fluorescence moieties such as rhodamine B extends their usefulness as imaging agents into the visible wavelength region and enables their use at concentrations as low as 10 μg/mL. The multi-functionalized CDs have superior photostability compared to known mitochondrial probes, cell compatibility for stable long-term mitochondrial imaging and apoptotic marker potential. Their uptake is dependent on mitochondrial membrane potential Δψ_m_ and this induces preferential localization in malignant cells. This renders them suitable for Δψ_m_ cell studies and promising vectors for the mitochondria-targeted delivery of anti-cancer drugs.

## Data Availability

All data have been present in main text and supplementary materials.

## Supplementary Materials

The following are available online at www.mdpi.com/article/10.3390/ph14090932/s1, Table S1: Quantum yields of CDs obtained employing a molar ratio of 1:1 CA:EDA microwave-irradiated for different time periods, Table S2: Quantum yields of CDs obtained for various CA:EDA molar ratios after microwave irradiation for 2 min, Table S3: Alkyl-TPP-functionalized carbon dots with various moles of C_4_TPP or C_10_TPP, their number of primary amino groups and the degree of functionalization for each sample, Figure S1: ^1^H- and ^13^C-NMR spectra of N-doped CDs, Figure S2: ^1^H-NMR spectra of alkyl-TPP-functionalized CDs, Figure S3: FTIR spectra of N-doped and TPP-functionalized CDs, Figure S4: TEM and high-resolution TEM images of N-doped CDs, Figure S5: X-ray diffractogram of N-doped CDs, Figure S6. Fluorescence intensity of N-doped CDs in water vs. pH, Figure S7: Quenching of the fluorescence of N-doped CDs by Fe(II) and Fe(III) ions in water, Figure S8: Fluorescence excitation and emission spectra of CD-C_4_TPP•Rh, Figure S9: UV–Vis spectra of rhodamine B, CD•Rh and its alkyl-TPP derivative CD-C_4_TPP•Rh, Figure S10: UV–Vis spectra of TPP-C_4_-COOH and of TPP-C_10_-COOH, Figure S11: Cytotoxicity of alkyl-TPP-functionalized carbon dot derivatives in MDA-MB-231 cell line.
