# Supplementary material for: Engineering Mitochondriotropic Carbon Dots for Targeting Cancer Cells
Source: Pharmaceuticals (Basel). 2021 Sep 16;14(9):932. doi: 10.3390/ph14090932 (PMC8470554; doi:10.3390/ph14090932)
Supplement: Supplementary file 1 [file pharmaceuticals-14-00932-s001.zip › pharmaceuticals-1377331-supplementary.pdf]

pharmaceuticals

MDPI

Article

Engineering Mitochondriotropic Carbon Dots for Targeting Cancer Cells

Archontia Kaminari [1], Eleni Nikoli [1], Alexandros Athanasopoulos [2], Elias Sakellis [1], Zili Sideratou [1] and Dimitris Tsiourvas [1,*]

[1] National Centre for Scientific Research "Demokritos", Institute of Nanoscience and Nanotechnology, 15310 Aghia Paraskevi, Greece; a.kaminari@inn.demokritos.gr (A.K.); h.nikoli@inn.demokritos.gr (E.N.); e.sakellis@inn.demokritos.gr (E.S.); z.sideratou@inn.demokritos.gr (Z.S.)

[2] National Centre for Scientific Research "Demokritos", Institute of Biosciences and Applications, 15310 Aghia Paraskevi, Greece; alexandr@bio.demokritos.gr

* Correspondence: d.tsiourvas@inn.demokritos.gr; Tel.: +30-210-650-3616

Supplementary Material

Table S1. Quantum yields of CDs obtained employing a molar ratio of CA:EDA = 1:1 and microwave irradiated for different time periods (1.5 to 4.0 min).

CA		EDA		NH_2/COOH Ratio	Time	QY
mass (g)	mmol	V (μL)	mmol		min	(%)
1.00	5.21	350	5.21	0.667	1.5	39.4
1.00	5.21	350	5.21	0.667	2.0	46.2
1.00	5.21	350	5.21	0.667	3.0	40.3
1.00	5.21	350	5.21	0.667	4.0	21.6

Table S2. Quantum yields of CDs obtained for various CA:EDA molar ratios after microwave irradiation for 2 min.

CA		EDA		NH_2/COOH Ratio	CA:EDA Molar Ratio	QY
mass (g)	mmol	Volume (μL)	mmol			(%)
1.00	5.21	280	4.20	0.54	1.24	37.97
1.00	5.21	315	4.72	0.60	1.10	37.35
1.00	5.21	350	5.21	0.67	1.00	39.13
1.00	5.21	385	5.77	0.74	0.90	48.54
1.00	5.21	420	6.29	0.81	0.83	40.56

Table S3. Alkyl-TPP functionalized carbon dots synthesized by the interaction of carbon dots (100 mg) with various moles of C_4TPP or C_{10}TPP, the number of their primary amino groups and the degree of functionalization for each sample.

Sample	Alkyl-TPP (mmol)	Primary Amino Groups (mmol NH_2/g)	Degree of Functionalization (%)
CD		1.18	-
CD-C_4TPP-1	10	0.81	31 %
CD-C_4TPP-2	15	0.49	58 %
CD-C_4TPP-3	25	0.21	82 %
CD-C_{10}TPP-1	10	0.86	27 %

CD-C_{10}TPP-2	15	0.59	50 %
CD-C_{10}TPP-3	25	0.20	83 %

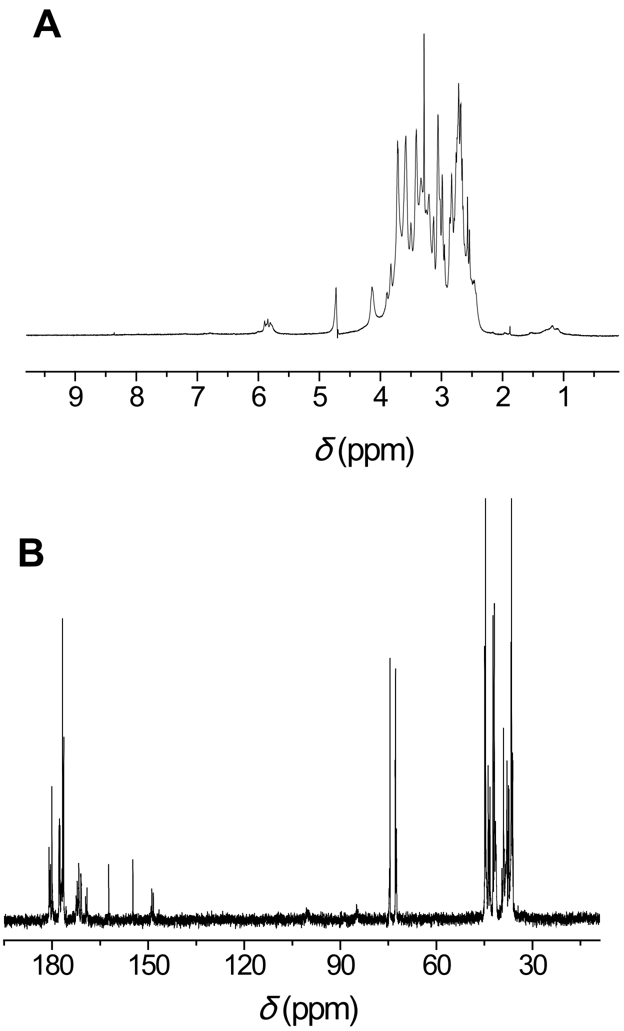

Figure S1. ^{1}H- NMR (**A**) and ^{13}C-NMR (**B**) spectra of N-doped CDs

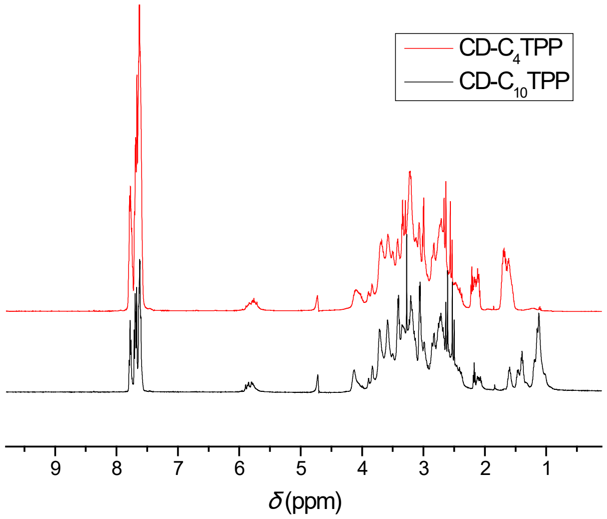

Figure S2. ^{1}H- NMR spectra of alkyl-TPP functionalized CDs

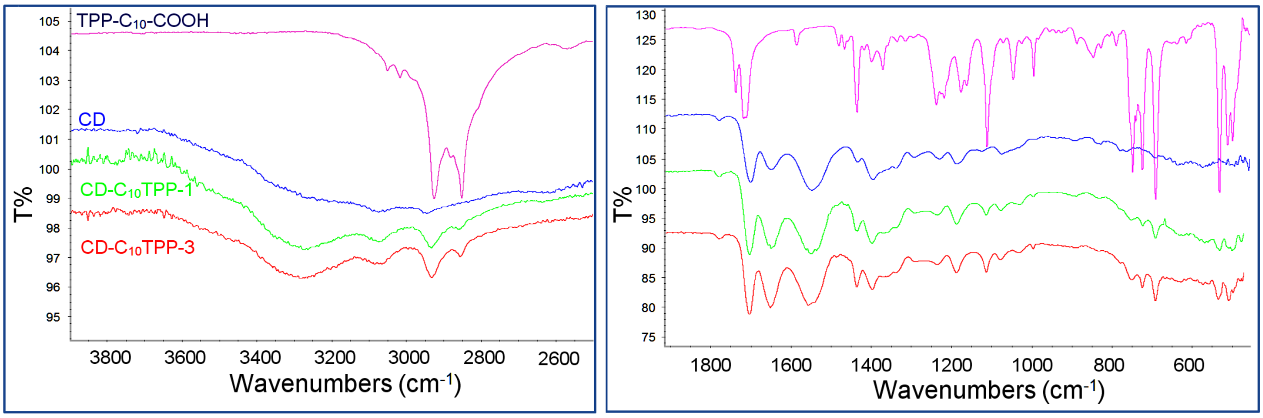

Figure S3. FTIR spectra of N-doped CDs, of (10-carboxydecyl)triphenylphosphonium bromide (TPP-C_{10}-COOH), and of C_{10}TPP functionalized CDs with two different degrees of functionalization, i.e., 27 and 83 % for CD-C_{10}TPP-1 and CD-C_{10}TPP-3, respectively.

A B

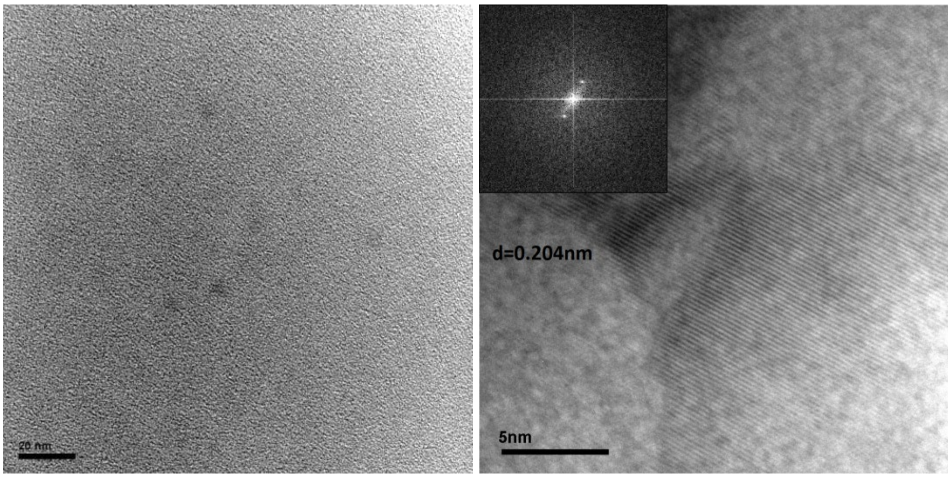

C D

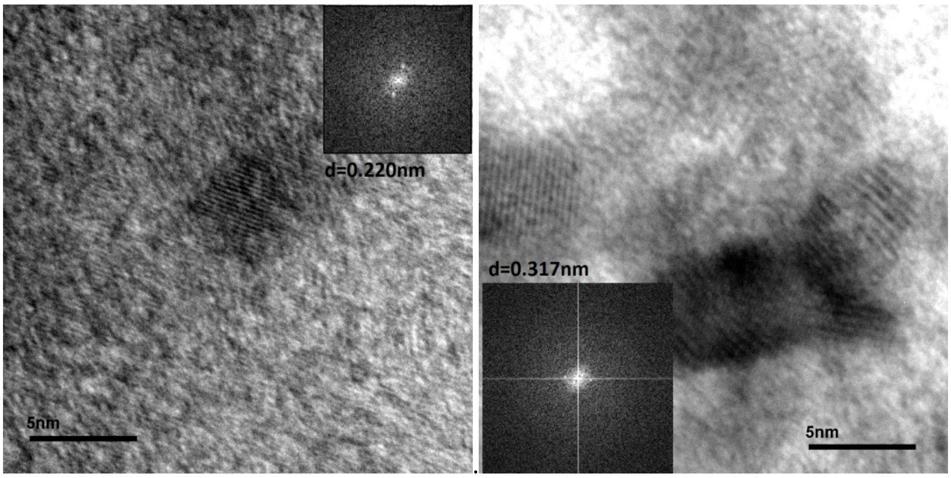

Figure S4. (**A**) TEM images of N-doped CDs. The size of the bar at the lower left corner is 20 nm. (**B**, **C**, **D**) High resolution TEM (HRTEM) images of N-doped CDs. The insets are the corresponding Fast Fourier transform (FFT) patterns.

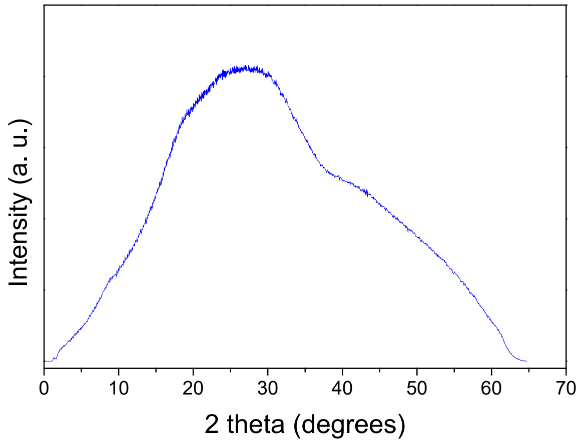

Figure S5. X-ray diffractogram of N-doped CDs.

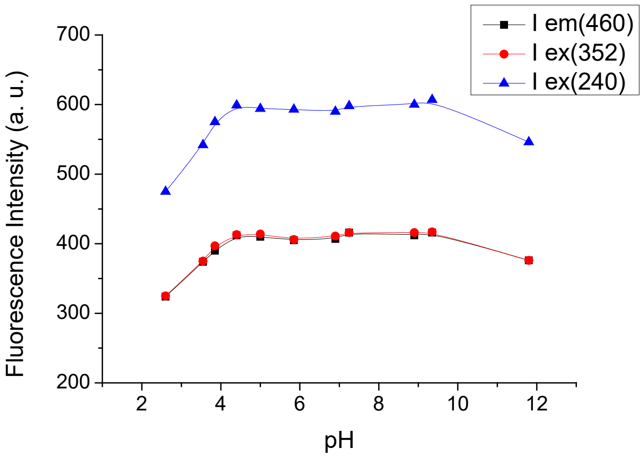

Figure S6. Fluorescence intensity of N-doped CDs in water vs. p*H*: Intensity of the emission spectra at 460 nm (λ_{ex}=352 nm) and of the excitation spectra at 240 and 352 nm (λ_{em}=460 nm).

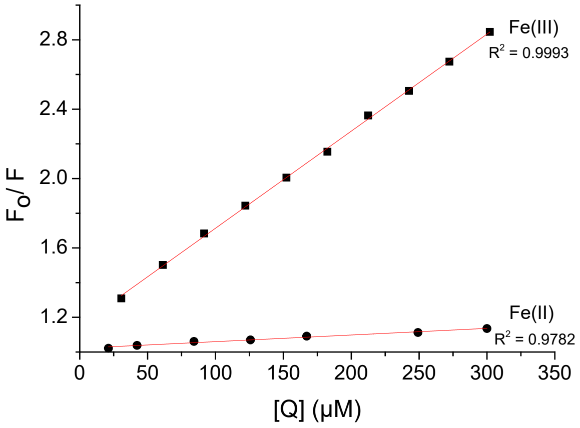

Figure S7. Quenching of the fluorescence of N-doped CDs by Fe(II) and Fe(III) ions in water. F_o and F are the fluorescence intensities in the absence and presence of quencher, respectively; while Q is the concentration of quencher.

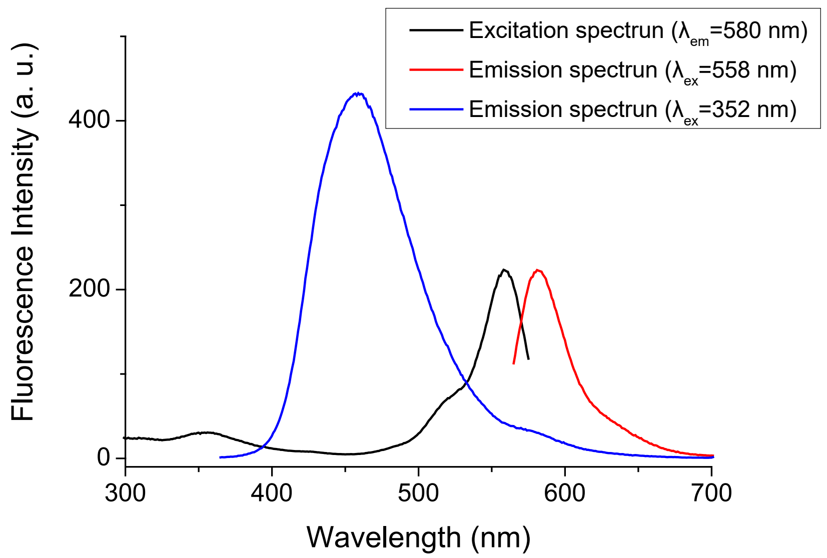

Figure S8. Fluorescence excitation and emission spectra of CD-C_4TPP•Rh (0.1 mg/mL).

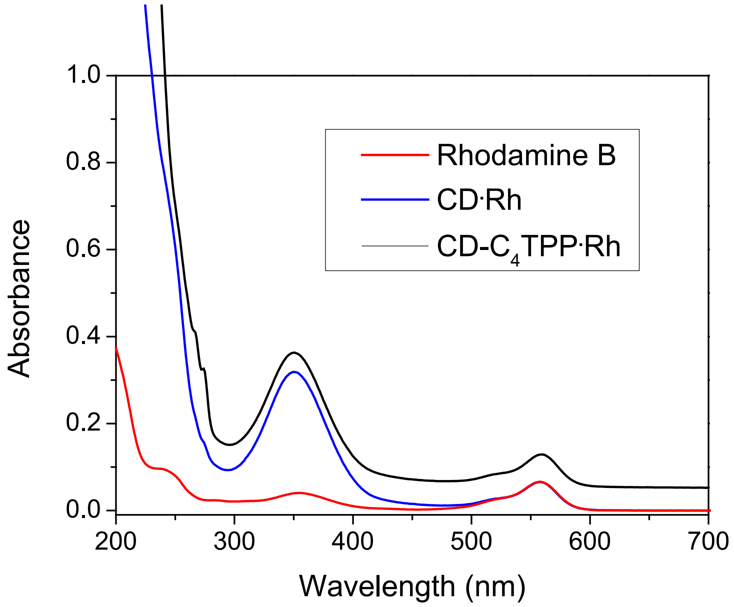

Figure S9. UV-Vis spectra of Rhodamine B, CD•Rh and its alkyl-TPP derivative CD-C_4TPP•Rh (0.1 mg/mL). For clarity reasons, the CD-C_4TPP•Rh curve is shifted by 0.05 units along the Y axis for clarity.

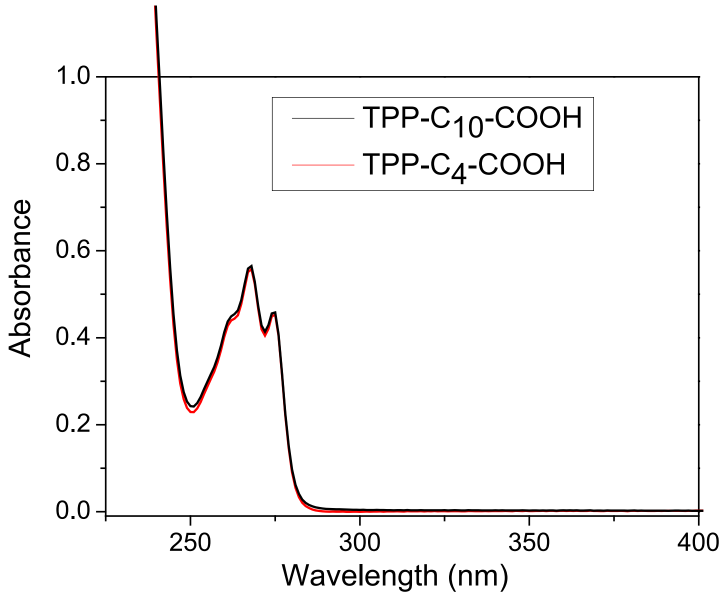

Figure S10. UV-Vis spectra of (4-carboxybutyl)triphenylphosphonium bromide (TPP-C_4-COOH; 0.2 mM) and of (10-carboxydecyl)triphenylphosphonium bromide (TPP-C_{10}-COOH; 0.2 mM) in ethanol.

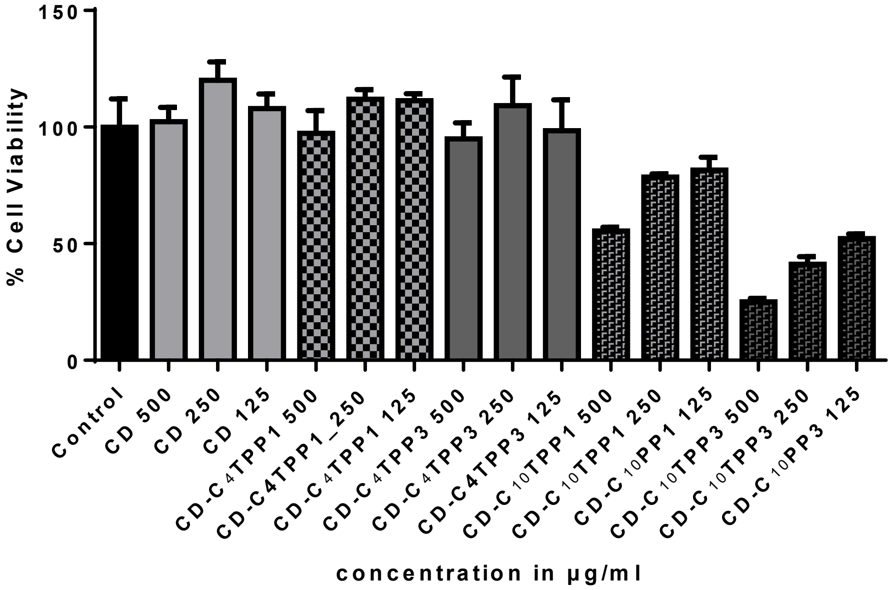

Figure S11. Cytotoxicity of alkyl-TPP functionalized carbon dots derivatives in MDA-MB-231 cell line. Cells were treated for 24 h with increasing concentrations (125-500 µg/mL) of CD-C_4TPP1, CD-C_4TPP3, and CD-C_{10}TPP1, CD-C_{10}TPP3 each of different degrees of functionalization (see Table S3). Cell viability was measured with MTT. Results are expressed as the mean ± standard error for at least three independent experiments.
